# Targeting cancer stem cells by using chimeric antigen receptor-modified T cells: a potential and curable approach for cancer treatment

**DOI:** 10.1007/s13238-017-0394-6

**Published:** 2017-03-13

**Authors:** Yelei Guo, Kaichao Feng, Yao Wang, Weidong Han

**Affiliations:** 1 Molecular & Immunological Department, Chinese PLA General Hospital, Beijing, 100853 China; 2 Bio-therapeutic Department, Chinese PLA General Hospital, Beijing, 100853 China

**Keywords:** cancer stem cells, chimeric antigen receptor, immunotherapy, translational medicine, response evaluation criterion

## Abstract

Cancer stem cells (CSCs), a subpopulation of tumor cells, have self-renewal and multi-lineage differentiation abilities that play an important role in cancer initiation, maintenance, and metastasis. An accumulation of evidence indicates that CSCs can cause conventional therapy failure and cancer recurrence because of their treatment resistance and self-regeneration characteristics. Therefore, approaches that specifically and efficiently eliminate CSCs to achieve a durable clinical response are urgently needed. Currently, treatments with chimeric antigen receptor-modified T (CART) cells have shown successful clinical outcomes in patients with hematologic malignancies, and their safety and feasibility in solid tumors was confirmed. In this review, we will discuss in detail the possibility that CART cells inhibit CSCs by specifically targeting their cell surface markers, which will ultimately improve the clinical response for patients with various types of cancer. A number of viewpoints were summarized to promote the application of CSC-targeted CART cells in clinical cancer treatment. This review covers the key aspects of CSC-targeted CART cells against cancers in accordance with the premise of the model, from bench to bedside and back to bench.

## Introduction

Cancer stem cells (CSCs), a small population of tumor cells first described in acute myeloid leukemia (AML), have been identified in various types of solid tumors, including liver, gastric, brain, breast, and prostate, and could promote tumorigenesis, metastasis, and relapse because of their self-renewal and differentiation capacity (Lapidot et al., 1994; Bonnet and Dick, 1997; Ma et al., 2007; Fukuda et al., 2009; Hemmati et al., 2003; Al-Hajj et al., 2003; Collins et al., 2005; Gupta et al., 2009). Although current anti-cancer treatments such as chemo- and radio-therapy are effective in eliminating most tumor cells, tumor relapse and/or metastasis is still a high risk for patients due to the insensitivity of CSCs to these conventional therapies, with new tumors initiating from the remaining CSCs (Reya et al., 2001; Hong et al., 2015). In addition, CSCs are greatly correlated to the clinical response of malignancies, which results in a poor prognosis and a short survival time when the tumor tissue contains a high ratio of CSCs (Frank et al., 2010). Altogether, an effective approach to specifically eliminate CSCs is urgently required to improve the clinical response of cancer treatment.

Recently, a new treatment strategy using chimeric antigen receptor-modified T (CART) cells has shown unprecedented clinical outcomes in hematologic malignancies, and its safety and feasibility in solid tumors was confirmed (Wang et al., 2014; Dai et al., 2015; Wang et al., 2015; Ahmed et al., 2015; Lamers et al., 2013; Feng et al., 2016). It is well known that CART cells can specifically target tumor cells by expressing a chimeric antigen receptor (CAR) constructed with an extracellular binding domain of a single-chain fragment of the antibody variable region (scFv) and the intracellular signaling domains of CD3zeta, coupled with or without co-stimulatory molecules such as CD28 and CD137. Among their properties, specific tumor recognition ability and improvement of T cell activation, proliferation, and survival are responsible for the effectiveness of anti-tumor treatment.

On the basis of the information mentioned, in theory, targeting CSCs by using CART cells may be an effective cure strategy for cancers. CART cells can specifically recognize antigens expressed on the cell surface of CSCs, and several potential antigens have been identified, such as CD44, CD90, CD133, aldehyde dehydrogenases (ALDH), and epithelial cell adhesion molecule (EpCAM) (Hong et al., 2015). Thus far, several attempts using CART cells against CSCs have been tested in several types of solid tumors in animal models, including anti-CD133 CART cells in treating glioblastoma, and anti-EpCAM CART cells for prostate cancer and peritoneal carcinomatosis from gastrointestinal and gynecologic malignancies (Zhu et al., 2015; Deng et al., 2015; Ang et al., 2017). In addition, a case report was published on advanced cholangiocarcinoma treated with anti-EGFR CART cells combined with anti-CD133 CART cells (Feng et al., 2017). However, the research has just started, and there is more work that needs to be implemented. To improve the clinical response for cancers, the development of CART cells for CSCs is imperative.

Therefore, in this review, we will briefly describe the characteristics of CSCs and critically discuss the possibility that CART cells targeting CSCs increase the clinical efficacy for various cancers. This review summarizes the key viewpoints to state the potential and feasibility of CSC-targeted CART cells for cancer treatment.

## Possibility of CSCs as a Therapeutic Target for Cart Cells

### Concise review of the identification and characteristics of CSCs

In bulk tumors, there is a small population of tumor cells that have the ability to self-renew and differentiate; thus, inducing the current conventional anti-cancer therapies cannot fully eliminate tumor cells (Lapidot et al., 1994). These self-renewing cells are called CSCs, and they play an important role in tumorigenesis, metastasis, and relapse. CSCs were first found in a leukemic cell transplanted mouse model in the 1960s (Bruce and Van Der Gaag, 1963). At that time, a detailed description of CSCs in AML was reported, in which a population of CD34^+^CD38^−^ had the ability to self-renew and differentiate (Bonnet and Dick, 1997). Subsequently, CSCs were identified in various types of solid tumors, including liver, gastric, brain, breast, prostate, and some other tumors (Bonnet and Dick, 1997; Ma et al., 2007; Fukuda et al., 2009; Hemmati et al., 2003; Al-Hajj et al., 2003; Collins et al., 2005; Gupta et al., 2009). On the basis of the specific features, CSCs are correlated to cancer evolution, and the CSC model has been suggested to be one of the tumor progression models (Dragu et al., 2015).

### Current strategies of targeting CSCs in cancer treatment

In the past several decades, cancer has become the major cause of death worldwide compared with other diseases (Stewart et al., 2014). Conventional therapies including surgery, chemo- and radio-therapy have indicated promise and efficacy for treating cancers, but ultimately resulted in treatment resistance and cancer relapse due to CSC-specific features. Thus, a strategy to effectively eradicate CSCs is needed to improve the clinical outcome for various cancers. Because CSCs express specific cell surface markers, the idea that specifically killing CSCs with their antibodies or other products can induce unexpected effects. Recent attempts to target CSCs have indicated that the approach can effectively improve the clinical response for cancers. For example, a study reported that the antibody CD44, one of the most established and common surface markers of CSCs, can suppress tumor progression and cause apoptosis of leukemic cells (Liu and Jiang, 2006; Song et al., 2004). Another study reported that *in vitro* proliferation and *in vivo* tumor growth of CD133-positive cancer cells could be inhibited by the CD133 antibody conjugated with drugs (Smith et al., 2008). A further study of CD133 in colorectal cancer (CRC) treated with asymmetric bispecific antibody (BiAb) consisting of CD133 and CD3 antibodies indicated strong anti-tumor efficacy (Zhao et al., 2015). In addition, interestingly, a number of reports suggested that a CSC-specific antibody-incorporated liposomal nanoparticle delivery system loaded with drugs or a suicide gene could significantly improve anti-tumor ability in solid tumors (Wang et al., 2012; Jain and Jain, 2008; Jain et al., 2010). Among these available studies, the approach of targeting CSCs is promising and effective for treating cancers.

### The possibility for tumor treatment using CSC-targeted CART cells

The risk of relapse and treatment resistance is the major problem of all recent cancer treatments. Taken together, searching for efficient approaches to improve the clinical response without severe toxicity is the ultimate purpose of tumor therapy. More recently, CART cells have shown great promise for treating various cancers, and they have become one of the indispensable strategies for tumor therapy. CART cells were first reported in the 1980s by the Eshhar group, and they can directly target tumor cells in an MHC-independent manner through expressing a CAR molecule, which consists of an extracellular antigen recognition domain (single-chain fragment of the variable region antibody), a transmembrane domain, and a cytoplasmic signaling domain (Gross et al., 1989; Kershaw et al., 2013; Sadelain et al., 2013). Over almost two decades, numerous studies have indicated success using CART cells in the treatment of hematologic malignancies. For example, CD19-redirected CART cells were used in patients with B-lineage cancer, including multiple myeloma, chronic lymphoid leukemia, acute lymphoid leukemia, and diffuse large B-cell lymphoma (DLBCL) (Dai et al., 2015; Garfall et al., 2015; Porter et al., 2011; Grupp et al., 2013; Kochenderfer et al., 2015). Further, anti-CD20 CART cells were used for non-Hodgkin lymphomas (NHL) and DLBCL (Wang et al., 2014; Till et al., 2012). In addition, the strategy of using CART cells has been indicated to be safe and feasible in treating solid tumors (Ahmed et al., 2015; Lamers et al., 2013; Feng et al., 2016). Even so, CART cell products need to optimize the improvement of clinical outcomes in the development of biotechnology. For example, to the best of our knowledge, the design of CART cells, including the selection of T-cell-activated signaling molecules and types of T cells, can induce different clinical results (Jensen and Riddell, 2015).

It is well known that CART cells can be successfully used for cancer treatment; however, the disadvantages must be studied clinically. Several advantages of CART cells, according to their features, include: (1) specific lysis; (2) duration *in vivo*; and (3) recognition of tumor cells in a MHC-independent manner. Nevertheless, the disadvantages may be also triggered by CART cell features. The disadvantages, including on-targeted/off-tumor toxicity, cytokine release syndrome (CRS), and soluble tumor syndrome, have a tremendous risk to patient health. Therefore, preclinical tests must be conducted before treating cancer patients with CART cells.

After careful consideration, the hypothesis regarding the treatment for CSCs using CART cells is feasible for cancer therapy based on the characteristics of CART cells and CSCs. Currently, the research on CART cells targeting CSCs is limited. To date, three studies using CSC-targeted CART cells have been reported in animal models (Zhu et al., 2015; Deng et al., 2015; Ang et al., 2017). A study has indicated that patient-derived glioblastoma stem cells can be killed by anti-CD133 CART cells both *in vitro* and in an orthotopic tumor model *in vivo* (Zhu et al., 2015). However, in this research, CART cells could be functionally impaired by CSCs, because CD57 was rapidly up-regulated on CART cells when they had direct contact with CD57-positive target cells. CD57 has been described as a marker associated with terminal or near-terminal T-cell differentiation (Strioga et al., 2011; Focosi et al., 2010; Wu et al., 2012). Another study of prostate cancer treatment by CART cells, specific for EpCAM, indicated some evidence of anti-tumor efficacy *in vitro* and in animal models (Deng et al., 2015). A further study using anti-EpCAM CART cells for local treatment of peritoneal carcinomatosis in xenograft mice demonstrated the possibility of this approach for the clinical treatment of gastrointestinal and gynecologic malignancies (Ang et al., 2017). Further, a case report on a patient with advanced cholangiocarcinoma treated with anti-EGFR CART cells combined with anti-CD133 CART cells indicated the safety and feasibility of clinical cancer treatment with CSC-targeted CART cells (Feng et al., 2017). The data from these tests suggest that the adoptive transfer of CSC-specific CART cells is a potential and promising treatment for cancers.

### The preclinical evaluation of CSC-targeted CART cell therapy

Before using CSC-targeted CART cells for clinical cancer treatment, the effectiveness of these cells needs to be evaluated. The surface expression of CAR molecules and, specifically, cytolytic activities *in vitro* must be studied. To better evaluate CSC-targeted CART cells, xenograft models are important to test their anti-tumor activity *in vivo*. Recent reports suggest that cell line-derived xenograft (CDX) and patient-derived xenograft (PDX) models represent preclinical efficacy models in oncology, whereas studies conducted with PDX are more predictive of clinical outcome than those with CDX (Rosfjord et al., 2014; Julien et al., 2012). In addition, studies in which patient’s fresh tumor tissues are transplanted into immunodeficient mice offers possibilities for preclinical evaluation of new cancer therapies (Julien et al., 2012). In this proof-of-concept testing using CSC-targeted CART cells to treat cancer, PDX may be a feasible animal model to test the effectiveness of CART cells specifically eradicating CSCs, providing predictive data to establish the anti-tumor activity of CSC-targeted CART cells in clinical cancer treatment.

## Potential Target Antigens of CSCS for Cart Cells

CART cells can specifically recognize tumor cells and inhibit their growth and proliferation, suggesting that cell surface markers expressed on CSCs probably provide potential targets for CART cell-based immunotherapy. Numerous studies have indicated that various surface markers (Table 1) such as CD133, CD90, ALDH, and EpCAM, are used to identify and isolate CSCs in cancer types, and that their expression levels are different from those of other bulk tumor cells (Zhu et al., 2015; Deng et al., 2015; Pan et al., 2015). Therefore, these markers could be important target antigens for CART cells in cancer treatment, making these genetically modified cells specifically eliminate CSCs and inhibit tumor relapse and metastasis.

**Table 1:** Cell surface markers express on CSCs

Marker	Cancer types
CD133	Brain, lung, liver, gastric, colorectal, and ovarian
CD90	Brain, breast, lung, liver, pancreatic, and esophageal
CD47	AML, NHL, ALL, MM, brain, breast, colon, ovarian, and bladder
CD44	Head and neck, breast, lung, liver, pancreatic, gastric, colorectal, bladder, cervical, ovarian, and prostate
CD24	Head and neck, breast, lung, liver, pancreatic, and colorectal
ALDH	AML, MM, brain, breast, lung, liver, pancreatic, gastric, colorectal, and ovarian
EpCAM	Breast, liver, pancreatic, colon, and prostate

AML, acute myeloblastic leukemia; ALDH, aldehyde dehydrogenase; ALL, acute lymphoblastic leukemia; EpCAM, epithelial cell adhesion molecule; MM, multiple myeloma; NHL, non-Hodgkin lymphoma

### CD133

CD133, a five-transmembrane glycoprotein that was first found as a surface marker that localized at membrane protrusions of CD34^+^ hematopoietic stem cells, has been widely used to isolate CSCs from various tumors (Shmelkov et al., 2005; Yin et al., 1997; Bidlingmaier et al., 2008). It has now been confirmed to be highly expressed in many cancer types, including brain, lung, liver, gastric, colorectal, and ovarian (Yi et al., 2008; Alamgeer et al., 2013; Yamashita and Wang, 2013; Hibi et al., 2010; Zhang et al., 2014; Baba et al., 2009). Unfortunately, it is not yet clearly known whether the cellular stemness in other cancer types is maintained by the downstream intracellular signaling of CD133 (Su et al., 2015). Clinical studies suggested that CD133 subpopulation in cancers has a positive correlation with treatment resistance and poor prognosis (Dragu et al., 2015; Zhang et al., 2010). Therefore, CD133 could be a potential target for CSC treatment. Recently, numerous studies have indicated that the strategies for CSC treatment by targeting CD133, such as polymeric nanoparticles loaded with paclitaxel and anti-CD133 antibodies, have been formed to effectively kill CSCs (Swaminathan et al., 2013; Skubitz et al., 2013). On the basis of these anti-CD133 CSC therapies, CART cells targeting surface marker CD133 can probably effectively eliminate CSCs. However, thus far, there is only one study that has used CD133-specific CART cells to treat patient-derived glioblastoma stem cells (Zhu et al., 2015).

### CD90

CD90, another most important surface marker of CSCs, has been found in brain, breast, lung, liver, pancreatic, and esophageal cancer types (Woo et al., 2015; Wang et al., 2015; Khan and Mukhtar, 2015; Sukowati et al., 2013; Zhu et al., 2014; Tang et al., 2013). It is a glycophosphatidylinositol-anchored glycoprotein that plays a key role in cell-to-cell and cell-to-matrix interactions (Rege and Hagood, 2006). Similar to CD133, CD90 also plays a role in self-renewal, growth, and differentiation of CSCs, and is an important regulatory factor of oncogenesis in many malignant diseases (Sukowati et al., 2013). Concerning the surface marker of CSCs, CD90 is correlated with tumor aggression and poor prognosis (Sukowati et al., 2013; Lingala et al., 2010; Lu et al., 2011). A recent study indicates that anti-CD90 therapy, using its antibody-mediated, water-soluble CdSe core nanocrystals loaded with photosensitizers, specifically killed CD90-positive leukemia CSCs (Bakalova et al., 2004). Therefore, it is reasonable that targeting CD90-CSCs by CART cells is a therapeutic treatment for many cancers.

### ALDH

On the basis of accumulating evidence, ALDH, defined as a superfamily of enzymes that participate in the metabolism of aldehyde derivatives, has been used as a specific biomarker to identify CSCs in a large number of cancers (Ginestier et al., 2007; Feldmann et al., 2007; Marchitti et al., 2008). The high activation of ALDH was correlated with enhanced tumorigenicity and chemoresistance (Ginestier et al., 2007). ALDH was used as an indicator of poor outcome in patients with breast cancer (Ginestier et al., 2007). A recent study reported that the elimination of CSCs with ALDH-specific CD8^+^ T cells could decrease the spontaneous burden of pancreatic and breast cancers *in vivo* (Visus et al., 2011). Thus far, ALDH has been suggested as a valid target for cancer treatment using immunotherapy, particularly CART cell-based therapy.

### EpCAM

It is well known that EpCAM is more widely expressed on CSCs, and it is also regarded as a tumor-associated antigen (TAA) (Munz et al., 2009). Its expression in the apical surface of tumor cells was remarkable, but minimal in the basolateral surface of normal epithelial cells (Ogura et al., 1998; Salomon et al., 2014). Recent studies have established that EpCAM is overexpressed on CSCs from several cancer types, including breast, colon, pancreas, and prostate tumors (Bakalova et al., 2004; Gires et al., 2009; Li et al., 2007; O’Brien et al., 2007; Ricci-Vitiani et al., 2007). EpCAM plays an important role in self-renewal, proliferation, and differentiation of CSCs; moreover, its high-level expression can increase tumorigenesis in breast, colon, and head and neck squamous cell carcinoma (Van der Gun et al., 2010; Visvader and Lindeman, 2008) as well as migration and invasion of cancers, such as breast cancer and retinoblastoma (Mitra et al., 2010; Osta et al., 2004). Accordingly, EpCAM has been considered to be a potential therapeutic target to treat cancer. Several approaches such as anti-EpCAM antibodies and synthetic oligonucleotide were generated to target cancer (Schmidt et al., 2010; Shigdar et al., 2011; Song et al., 2013). Interestingly, CART cells were developed to target CSC-antigen EpCAM to eliminate prostate cancer (Deng et al., 2015), demonstrating that EpCAM-specific CART cells had tremendous therapeutic potential for cancer treatment.

### Additional CSC identified targets

In addition to CD133, CD90, ALDH, and EpCAM, there are several additional CSC identified target antigens. To the best of our knowledge, various CD cell surface markers have been used to identify and isolate CSCs in human cancer cells. For example, CD47 is a transmembrane protein that serves as a cell surface receptor for signal regulatory protein-alpha and secreted matricellular protein thrombospondin-1 (Naujokat, 2012). It was detected in nearly all malignancies, including NHL, acute lymphocytic leukemia (ALL), AML, glioblastoma, ovarian cancer, and colon cancer, and was found to control cell survival and growth (Majeti et al., 2009; Willingham et al., 2012; Chao et al., 2011a, b; Kim et al., 2012). Moreover, high expression levels of CD47 were used to predict a poor clinical outcome in some solid tumors (Willingham et al., 2012). Preclinical studies have demonstrated that the strategy of using monoclonal antibody (mAb) specific for CD47 could inhibit growth and metastasis of tumor cells for cancer treatment (Willingham et al., 2012). B6H12 and B6H12.2, two anti-CD47 mAbs, could effectively prevent the growth of glioblastoma, ovarian cancer, breast cancer, leiomysarcoma, and AML in xenograft mice models (Majeti et al., 2009; Willingham et al., 2012; Edris et al., 2012). Therefore, CD47 is a potential therapeutic target for tumor therapy. In addition, CD44 and CD24 have recently been identified as CSCs from almost all tumors (Hong et al., 2015; Chen et al., 2015). The expression of CD44 and CD24 is closely correlated with tumorigenesis, tumor progression, metastasis, and chemotherapy resistance (Chen et al., 2015). Recent studies indicated CD44 and CD24 are therapeutic targets for the treatment of various tumors (Hong et al., 2015; Ma et al., 2015; Zhang et al., 2014). In conclusion, one of these CD cell surface markers, CD47, CD44, or CD24, can be an attractive new target for the elimination of CSCs by CART cells in multiple cancer types.

### The principles of CSC antigen selection for CART cell therapy

Many cell surface antigens presenting on CSCs have been reported, and they vary according to tumor type (Table 1). Although a better understanding of CSC surface markers was contributed recently, more work is needed to complete the picture. The cell surface markers of CSCs are often observed differently from other cell surface markers, and normal cells also express these CSC surface markers (Dragu et al., 2015). Therefore, antigen selection is extremely important for CSC-targeted CART cell therapy. The principles of CSC antigen selection should follow these guidelines: (1) selection of a high level of antigen expression, aiming to clear CSCs to prevent tumor relapse; (2) selection of highly specific CSC antigen to avoid damaging normal cells that express the same antigens; and (3) selection of the same antigens that expressed on different tumor types, because this will benefit the development of universal CSC-targeted CART cells. Moreover, new CSC surface antigens may be detected in the future. Large sample analyses need to implemented in various tumor types to capture the new surface antigens that have CSCs features.

## Potential Toxicity of CSC-Targeted Cart Cells

The safety in clinical trials is vitally important for research participants and for those with malignancies receiving CSC-targeted CART cell treatment. The major difficulty and challenge for CART cells targeting CSCs is the development of on-target/off-tumor toxicity that is caused by CART cells killing normal cells, because CSC surface markers are also found on normal cells. This toxicity had been reported in previous studies. For example, durable B-cell aplasia was caused after the administration of anti-CD19 CART cells because of their robust expansion (Maude et al., 2014). In another trial, a patient with metastatic colon cancer who received lymphodepletion chemotherapy, followed by treatment with ERBB2-specific CART cells combined with IL-2, developed acute respiratory distress syndrome and died 5 days after the treatment (Morgan et al., 2010). The study indicated that a large number of CART cells trafficked to the lung and targeted the lung epithelial cells, which expressed a low level of ERBB2. This led to the release of pro-inflammatory cytokines, which resulted in pulmonary toxicity and the death of the patient. For the potential antigens that are used for CART cells targeting CSCs, few are specific, such as CD133 expressed in normal brain tissues, hematopoietic stem cells, and endothelial progenitor cells, and ALDH expressed in hematopoietic stem/progenitor cells (Corbeil et al., 2010; Kastan et al., 1990). Therefore, there are significant safety challenges associated with the use of CSC-specific CART cells that are a concern and need to be resolved. It is concluded that strategies to control target-mediated toxicity need to be explored to enhance tumor specificity of CSC-targeted CART cells.

There have been numerous studies recently that have aimed to develop strategies for reducing on-target/off-tumor toxicity by controlling activity and proliferation of CART cells. One approach to control the toxicity is to encode suicide genes in CART cells to selectively eliminate them *in vivo* after their adverse events begin and become uncontrollable (Khaleghi et al., 2012; Budde et al., 2013). Another approach is to design bispecific CARs to enhance the tumor specificity of CART cells. Examples include the tandem CAR (TanCAR), one of the bispecific CARs which targets two TAAs simultaneously using a CAR molecule with two antigen recognition moieties joined in tandem (Grada et al., 2013). This approach can protect normal tissue and avoid the risk of immune escape by increasing tumor specificity of these cells (Yee et al., 2002). Moreover, several studies have indicated that a small molecule used as a “switch” can enable and indirectly improve control activity of CART cells (Kim et al., 2015; Wu et al., 2015). This novel concept may ultimately lead to the enhancement and safety of CART cells for cancer treatment. These strategies are also important for engineering CSC-targeted CART cells to enhance the safety in cancer treatment.

Adverse events other than on-target/off-tumor toxicity have been reported in several clinical studies of CART cells in treating tumors, for example, CRS (Kim et al., 2015). These toxicities, too, may be experienced in cancer treatment with CSC-targeted CART cells. It is known that the administration of tocilizumab (an anti-IL-6 antibody), targeted immunosuppressive agents, or steroid therapy enables improved control of CRS (Lee et al., 2014). Thus far, with the control of on-target/off-tumor toxicity and CRS, the safety of CSC-targeted CART cells will be much improved in cancer treatment.

## Application of CSC-Targeted Cart Cells in Translational Medicine for Cancer Treatment

CART cells, a recent hotspot of cancer therapy, have shown durable clinical responses in hematologic diseases, offering a novel vision for specific anti-CSC strategy in tumor treatment after careful consideration of pre-studies. Nevertheless, few clinical trials on CSC-targeted CART cells were reported in the last few decades. Therefore, more attention and work are needed to translate CSC-directed CART cells into clinical applications for tumor therapy.

**Figure 1:**
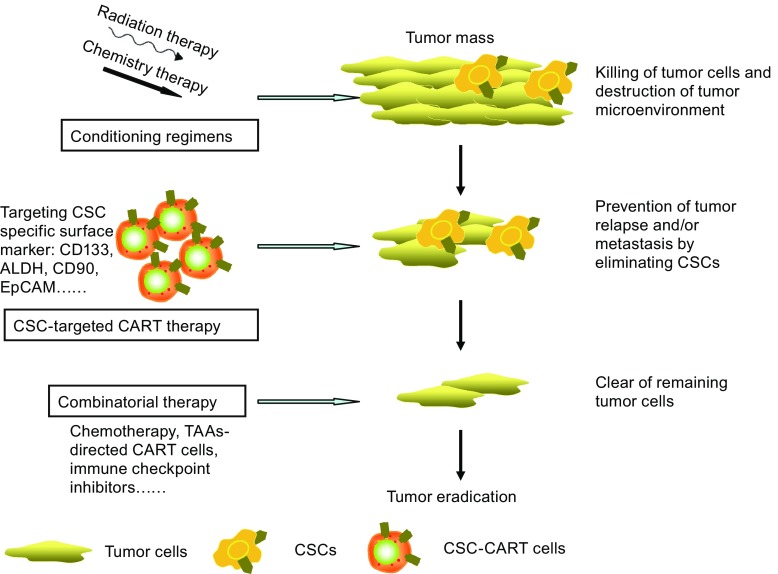
The potential roles of CSC-targeted CART cells in future cancer treatment. CART cells could effectively eliminate CSCs through targeting CSC-specific surface markers (CD133, ALDH, CD90, and EpCAM) to prevent tumor relapse and/or metastasis; furthermore, combinatorial therapies such as chemotherapy, radiotherapy, and immune checkpoint inhibitors could probably eradicate tumor cells to achieve a curable stage. ALDH, aldehyde dehydrogenases; CART, chimeric antigen receptor-modified T cells; CSCs, cancer stem cells; EpCAM, epithelial cell adhesion molecule; TAAs, tumor-associated antigens

The ultimate goal of cancer treatment is to be curative; moreover, theoretically, the strategy using CSC-targeted CART cells could be curative for tumors. However, this is faced with several barriers, such as tumor microenvironment that can inhibit immunotherapy. Tumor cells cannot be completely and potently eliminated using CSC-targeted CART cells alone. CART cells that are specific to CSCs combined with other therapies will be effective to enhance their anti-tumor efficacy (Fig. 1).

Several issues will be implemented for applying CSC-targeted CART cells to translational medicine in future tumor treatment, including the following aims: (1) to complete preclinical studies on CART cells treating tumors by targeting CSCs, for example optimal selection of CSC target antigen expressed on tumor cells, cytotoxicity of CART cells *in vitro* and in animal models, and monitoring and control of toxicities; (2) to perform the detailed protocol and to have it approved by a relevant ethics supervision department, and to register the clinical trials on the website www.clinicaltrials.gov; (3) to enroll appropriate patients to administer this specific anti-CSC strategy, and to monitor the outcomes and adverse events; (4) to optimize clinical protocol dependent on clinical response; and (5) to analyze the mechanism of CSC-targeted CART cells in tumor treatment from the basic study.

The field of cancer therapy by CART cells has rapidly developed, and more attention has been paid to it in recent years. Safety and efficacy of CART cells are critical for clinicians and patients, and current consistent and intensive practice from the bench to the bedside and back to bench could hardly improve the clinical outcome. For CART cells specific for CSCs, data from the first clinical trial of cancer therapy using this strategy are very important for future research, possibly achieving an unexpected outcome in accordance with the current protocol of CART cells for malignancies. However, few clinical trials concerning CART cells targeting CSCs registered on the website www.clinicaltrials.gov indicate that this cancer therapy is still in the exploration and development stage. More work, therefore, is needed to receive worldwide attention to strengthen the research of CART cells for CSCs. Fortunately, the group from Chinese PLA General Hospital had registered a clinical trial on the treatment of multiple types of advanced malignancies by using CSC marker CD133-directed CART cells on this website in 2015. Further, the first clinical trial with anti-CD133 CART cells from the group was reported, and indicated that the infusion of anti-CD133 CART cells was safe and feasible for cancer treatment (Feng et al., 2017).

## Clinical Response Evaluation Criteria of CSC-Targeted Cart Cells in Cancer Treatment

It is well known that response evaluation criteria in solid tumors (RECIST) and modified World Health Organization (WHO) criteria could be the typical criteria to evaluate antitumor responses of chemotherapeutic drugs. However, unfortunately, it seems that these two types of response evaluation criteria are not suitable for immunotherapies, due to the latter cytotoxicity on cancer cells with distinct characteristics in the clinic (Hoos et al., 2015). With the recent increase in immunotherapeutic strategies for cancer treatment, a unique response is needed for evaluation criteria to provide appropriate criteria for clinical outcome evaluation.

Immune-related response criteria (irRC), a novel criterion for immunotherapy in cancer treatment, was reported by Wolchok and colleagues in Clinical Cancer Research in 2009, and was designed to better evaluate the responses of immunotherapies (Wolchok et al., 2009). One of the most important differences between irRC and RECIST or WHO criteria is that irRC captures the responses in changes of all tumor lesions assessed from baseline, not only target lesions (Hoos et al., 2015; Wolchok et al., 2009; Ades and Yamaguchi, 2015). In addition, several unique characteristics of immunotherapies, including (1) appearance of new lesions, (2) delay in onset of clinical responses, (3) increase of tumor volume before tumor shrinkage, and (4) prolonged stable disease, can be detected by irRC (Wolchok et al., 2009; Hoos et al., 2010). Herein, irRC can help us to explain why 20% to 25% of patients with metastatic melanoma had durable survival after receiving ipilimumab, whereas RECIST or WHO criteria could not capture survival outcomes of patients as the new patterns of response rates (Wolchok et al., 2009; Hodi et al., 2010).

CSC-directed CART cell-based immunotherapy, as an anti-CSC strategy, could probably produce amazing clinical responses in future cancer treatment; nevertheless, there are no recent anti-CSC response evaluating guidelines to accurately assess patient outcome for this specific immunotherapy (Savona et al., 2015). The recent broad use of irRC has comprehensively captured responses with immunotherapies in clinical trials, and has shown that irRC could probably be a powerful tool to evaluate clinical responses, combined with either RESICT or WHO criteria (Hoos et al., 2015). This indicates that their concepts can be used to assess CSC-targeted CART cell responses in cancer treatment. Beyond this combined response evaluation pattern, several issues must be addressed, including (1) percentage of CSCs determined in biopsied target lesions by the immunohistochemistry method; (2) transgene copy numbers of CAR vectors in peripheral blood and biopsied lesions, monitoring *in vivo* persistence of CART-CSC cells; and (3) release of cytokines such as IL-6, IFN-gamma, and TNF-alpha, which assessed from baselines, will be also followed (Feng et al., 2016). Thus, these guidelines set the stage for a more accurate evaluation of clinical response in future cancer treatment with CSC-targeted CART cells.

Even so, the development of clinical response evaluation criteria is imperative to accurately present the effect on the patient after receiving CSC-targeted CART cells in cancer treatment. We would like to further update these guidelines according to the clinical response in future trials.

## Final Thoughts on CSC-Targeted Cart Cells in Cancer Treatment

Because of the characteristics of self-renewal and multi-lineage differentiation of CSCs, the efficacy of conventional cancer treatments including chemotherapy and radiotherapy is often low. The few attempts using CART cells to eliminate CSCs (e.g., using CART cells specific for CD133 or EpCAM for cancer therapy) (Zhu et al., 2015; Deng et al., 2015) indicated that the strategy of CART cells specific for CSCs can probably be regarded as a considerable treatment in various cancer types. Further, the impressive data generated from CART cells to treat liquid and solid tumors provide a novel concept for the use of CSC-targeted CART cells in cancer treatment. However, there is not enough available information to make a conclusion about the clinical response of CART-CSC cells in cancer treatment, although a number of *in vitro* and animal model studies suggest a therapeutic benefit. Unfortunately, these explorations of CART-CSC cells in cancer treatment are prohibited by ethics problems, whereas successful cancer treatment is only measured on patients in the clinic. Therefore, more detailed data about the preclinical effect of CART-CSC cells on various types of cancer will undoubtedly be in favor of the development of this therapy in future clinical trials.

The majority of currently available information on CSCs, in terms of their phenotypes, such as CD133, CD90, ALDH, and EpCAM, could act as targets for genetically modified T cells. To target these markers, CART cells could selectively eliminate CSCs, preventing tumor relapse and/or metastasis. However, currently, it remains unclear whether normal stem/progenitor cells could be damaged by CSC-targeted CART cells. Strengthening the basic research can accelerate CSC-targeted CART cells in cancer treatment and establish the safety and feasibility in the clinic, ensuring their downstream research applications. We believe that combined CSC-targeted CART cell therapy has great potential as a strategy in future clinical cancer treatment studies.
